# Circadian Regulation of Food-Anticipatory Activity in Molecular Clock–Deficient Mice

**DOI:** 10.1371/journal.pone.0048892

**Published:** 2012-11-07

**Authors:** Nana N. Takasu, Gen Kurosawa, Isao T. Tokuda, Atsushi Mochizuki, Takeshi Todo, Wataru Nakamura

**Affiliations:** 1 Laboratory of Oral Chronobiology, Graduate School of Dentistry, Osaka University, Osaka, Japan; 2 Theoretical Biology Laboratory, RIKEN Advanced Science Institute, Wako, Japan; 3 Department of Micro System Technology, Ritsumeikan University, Shiga, Japan; 4 Department of Radiation Biology and Medical Genetics, Graduate School of Medicine, Osaka University, Osaka, Japan; 5 Precursory Research for Embryonic Science and Technology, Japan Science and Technology Agency, Kawaguchi, Japan; University of Massachusetts Medical School, United States of America

## Abstract

In the mammalian brain, the suprachiasmatic nucleus (SCN) of the anterior hypothalamus is considered to be the principal circadian pacemaker, keeping the rhythm of most physiological and behavioral processes on the basis of light/dark cycles. Because restriction of food availability to a certain time of day elicits anticipatory behavior even after ablation of the SCN, such behavior has been assumed to be under the control of another circadian oscillator. According to recent studies, however, mutant mice lacking circadian clock function exhibit normal food-anticipatory activity (FAA), a daily increase in locomotor activity preceding periodic feeding, suggesting that FAA is independent of the known circadian oscillator. To investigate the molecular basis of FAA, we examined oscillatory properties in mice lacking molecular clock components. Mice with SCN lesions or with mutant circadian periods were exposed to restricted feeding schedules at periods within and outside circadian range. Periodic feeding led to the entrainment of FAA rhythms only within a limited circadian range. *Cry1^−/−^* mice, which are known to be a “short-period mutant,” entrained to a shorter period of feeding cycles than did *Cry2^−/−^* mice. This result indicated that the intrinsic periods of FAA rhythms are also affected by *Cry* deficiency. *Bmal1* ^−/−^ mice, deficient in another essential element of the molecular clock machinery, exhibited a pre-feeding increase of activity far from circadian range, indicating a deficit in circadian oscillation. We propose that mice possess a food-entrainable pacemaker outside the SCN in which canonical clock genes such as *Cry1*, *Cry2* and *Bmal1* play essential roles in regulating FAA in a circadian oscillatory manner.

## Introduction

The ability to anticipate periodic food availability is an exception to the SCN lesion-induced abolition of circadian rhythms [1], [2]. When food availability is restricted to a short temporal window in the day, animals display prefeeding locomotor activity and feeding-associated physiological changes known as “food anticipatory activity (FAA)”. The mechanism underlying these circadian rhythms is assumed to be a “food-entrainable circadian oscillation (FEO).” In this hypothesis, the FAA is driven by a FEO, which is entrained to the timing of periodic feeding. This hypothesis further posits that the FEO resides at a location different from that of the light-entrainable oscillator (the SCN, which is principally entrained to light/dark cycles) (for review, see [3], [4]).

The molecular framework of circadian oscillations is best studied in the SCN [5], [6]. A core set of circadian clock genes, such as *Period (Per1* and *Per2)*, *Cryptochrome (Cry1* and *Cry2)*, *Bmal1* and *Clock*, form autoregulatory transcription-translation feedback loops that are thought to drive daily rhythms in individual cells. Mutations of genes that are involved in these loops, either singly or in combination, can alter circadian rhythms, producing long-period, short-period or arrhythmic phenotypes [7]. These clock genes are expressed in a circadian manner not only in the SCN, but also in other parts of the brain and in many peripheral tissues [8], [9].

Under temporally restricted feeding (RF), clock gene expression in certain non-SCN brain regions shifts in conjunction with the emergence of FAA [10], [11], [12]. Certain clock gene mutations have been identified that affect the expression of FAA [13], [14]. Therefore, it is likely that known clock genes in one or more of those tissues is critical for generating FAA rhythms. On the other hand, mice lacking *Cry1/Cry2* [13], *Bmal1* [15], [16], [17] and *Per1/Per2* [17] retain FAA rhythms, although all are deficient in essential molecular clock components. These results suggest that FAA rhythms may not require the known molecular clock machinery, and that the temporal regulation of FAA rhythms might involve an entirely different clock, or even a non-circadian process.

The present study confirms the hypothesis that circadian oscillators regulate FAA rhythms in known molecular clock–deficient mice. We explored the range of periodicities to which FAA can be entrained by RF conditions [18], [19], [20]. Rats can show FAA to RF intervals within the circadian range (approximately 22–31 h) [18], [19], [20]. Because a very short period mutant mouse has been reported [21], we assumed that the lower limit of the circadian range is 20 h. Near the limits of entrainment, we expected to see relative coordination and/or free-run of FAA. Another possibility in molecular clock-deficient mice was to see no limitation of entrainment range, indicating that it is independent of circadian regulation. This would indicate the involvement of a canonical clock mechanism in the FEO.

## Materials and Methods

### Ethics Statement

This study was carried out in strict accordance with the law and notification of the Japanese government. The Protocol was approved by the Animal Care and Use Committee at Osaka University (permission#AD-20-042-0). All surgery was performed under isoflurane anesthesia, and all efforts were made to minimize suffering.

### Animals

*Bmal1^+/−^* mice [22], obtained from the Jackson Laboratory (Stock #009100), were intercrossed to generate wild-type and homozygous mutant mice. *Cry1* ^+/−/^ *Cry2* ^+/−^ mice [23] were backcrossed with the C57BL/6 strain for 10 generations and were intercrossed to generate single homozygous *Cry1* ^−/−^ and *Cry2* ^−/−^ mice. The mice were bred and group-housed in the Osaka University Graduate School of Dentistry animal facility in L:D 12∶12 with food and water available *ad libitum*.

### SCN Lesions

Mice were anesthetized with isoflurane and placed in a stereotaxic instrument (Narishige, Tokyo, Japan). The dorsal aspect of the parietal bones was exposed and cleaned. One hole was drilled around the bregma in the parietal bones. A lesioning electrode (tungsten needle coated with epoxy; 0.5 mm of the needle was exposed at the tip) was inserted into the brain and aimed at the SCN (0.4 mm posterior and ±0.25 mm lateral to the bregma, 5.7 mm depth from the skull surface). Lesions were generated with constant current (1 mA, 10 s; D.C. Lesion Making Device #53500, UGO Basile, Italy). Starting 1 week after surgery, locomotor activity was monitored using a passive infrared sensor or a running wheel under constant darkness (DD), and only mice showing arrhythmic behavioral periods for 3 weeks were selected for further studies. A chi-square periodogram was used to assess significant periodicities. At the end of the experiment, the brains were removed and sectioned at a 25-µm thickness through the extent of the lesion using a cryostat. Sections were stained with neutral red, and the locus of damage was determined via light microscope and a digital camera (Leica Microsystems, DFC290HD, Tokyo, Japan). Only mice with histologically verified complete SCN lesions were included in the data analysis.

### Activity Recording

Mice were housed individually in standard cages (182×260×128 mm) placed in light-tight, ventilated boxes. Locomotor activity was detected with a passive infrared sensor (Biotex, Kyoto, Japan) positioned 30 cm above the center of the cage floor. The sensor detected movement anywhere in the cage. Activity counts were monitored continuously by computer and summed and stored at 1-min intervals.

### Restricted Feeding

Before RF schedules, locomotor activity was monitored under *ad libitum* feeding conditions in DD for 7 days. During the first 24-h scheduled feeding, food was available for 8, 6 and 4 h on the first 3 days, respectively, with meal onset at a fixed time. Under RF conditions, food was provided and removed from the cage manually in constant darkness, with the aid of an infrared viewer to prevent light exposure. During *ad libitum* food access and food deprivation, doors of the isolation boxes were kept closed and the animals were not disturbed except during the exchange of food pellets.

### Data Analysis

ClockLab software (Actimetrics, USA) was used to analyze and display the activity data. A general feature of FAA is a steep onset of pre-feeding activity during a few hours immediately preceding meal time.

To estimate the pre-feeding activity phase, a circular analysis [24] was applied to serial activity data from individuals and group means of the mice. For individual analysis, each circular variable was interpreted as an angle 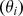 ranging from 0 to 360 degrees starting at the time of feeding, and the mean direction of activity was calculated for each cycle, resulting in the daily phase of activity. For group analysis, the same procedure was applied on the group mean variables of activity.

Activity data were quantified by calculating pre-feeding activity duration (in hours from the pre-feeding activity phase to meal time), and inter-day pre-feeding activity deviation (standard deviation of pre-feeding activity phase). FAA ratio was calculated by the fold change of mean locomotor activity in each mouse during the 2-h period prior to feeding compared with that of the rest of the day, except for the 4 h of time when food was available under RF conditions. This interval was omitted from the computations because of the direct activation and suppressive effect of food presentation on locomotor activity [17].

To estimate the phase and period of FAA rhythms during food deprivation, the cosinor method was used [25]. This technique estimates the cosine curve with a circadian periodicity that best fits the time series data in a 1-h bin and provides the coefficient of determination; R^2^.

Statistical significance between the two groups was determined by Student’s *t*-test. Statistical significance for three or more groups was determined by one-way ANOVA followed by Dunnett’s post-hoc analyses. All results are presented as mean ± SEM, and differences were considered significant at *P*<0.05.

## Results

### Limits of Entrainment to Periodic Feeding in Wild-type Mice with and without SCN

**Figure 1:**
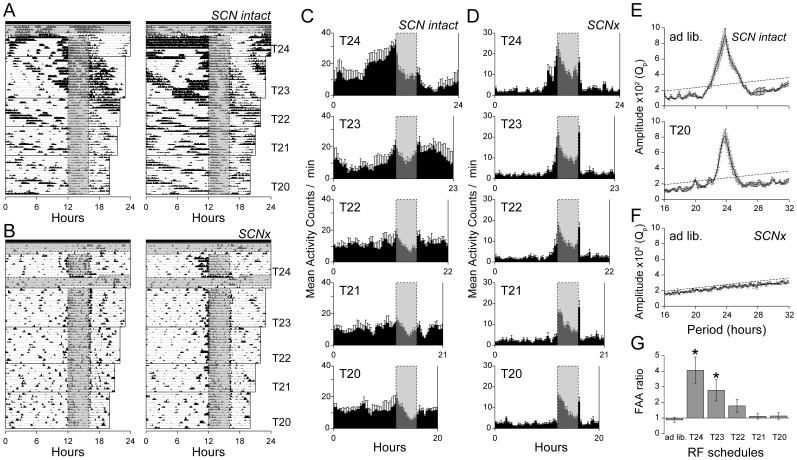
Limits of entrainment of FAA in wild-type mice. Representative actograms of locomotor activity plotted at a day length corresponding to each feeding schedule for easier visual assessment of the FAA in intact (**A**) and SCN-ablated (**B**) C57BL/6 wild-type mice. Time is plotted left-to-right in 10-min bins. Mice were maintained in constant darkness as indicated by closed bars at the top of the actograms. Feeding time is indicated by gray shaded boxes. (**C** and **D**) Mean profiles of locomotor activity are plotted along each RF schedule (**C**: SCN intact, n = 4; **D**: SCN ablated, n = 7). Mean profile was calculated using individual mean 7-day profiles. Each data point represents counts per minute averaged across a 20-min bin. Integrated periodogram of SCN-intact wild-type mice in baseline *ad libitum* feeding (**E**, top) and 20-h RF schedule (**E**, bottom) conditions (n = 4). Integrated periodogram of SCN-ablated wild-type mice in the baseline *ad libitum* feeding condition (**F**, n = 7). Significance level (dotted line) is *P* = 0.05 for individuals. (**G**) FAA ratios of the SCN-ablated mice on serial feeding schedules. Significant differences are indicated by asterisks (n = 7, vs. *ad libitum*, ANOVA post-hoc Dunnett test). *P<0.05.

Four wild-type mice with intact SCN were maintained in constant darkness and exposed to restricted feeding at periods (T-cycles) of 24, 23, 22, 21 and 20 h (Fig. 1A). The sequence of feeding conditions was as follows: food available *ad libitum* for 4 days, no food for 30 h, food available 4 h/day for 8 days (24-h RF), food available for the first 4 h every 23 h for 15 days (23-h RF), food available for the first 4 h every 22 h for 10 days (22-h RF), food available for the first 4 h every 21 h for 10 days (21-h RF), food available for the first 4 h every 20 h for 12 days (20-h RF). Before the start of the RF schedules, all mice exhibited free-running activity rhythms with periods ranging between 23.7 and 23.9 (Fig. 1E_ad lib.). These free-running rhythms were seen under the conditions of each T-cycle of RF without notable change in the free-running periods. Under the 24- and 23-h RF schedules, all mice showed robust FAA in locomotor activity (Fig. 1C). The actograms indicated high levels of FAA, although the effect was generally much more robust in the active phase of free-running rhythm than in the rest phase. In contrast to the 24- and 23-h schedule, the actograms indicated little or no anticipatory activity under the conditions of 22-, 21- and 20-h RF. Under these short T-cycles, the temporal rise of activity level occurred not before but right after food availability and gradually decreased during feeding. Periodogram analysis of the data for all 12 days with the 20-h RF schedule showed a major peak around 23.8 h, which corresponds to the free-running periods (Fig. 1E_T20). The minor periodogram peak corresponded to the 20-h RF schedule. This appeared to be the result of activity following feeding, which was synchronized with the food access period.

The results obtained with SCN-intact mice suggest that there are 3 rhythmic processes involved: free-running activity that is certainly driven by the SCN, direct driving of behavior by feeding, and FAA. Therefore, we next attempted to separate these processes by SCN lesioning, which is known to abolish free-running activity. By using mice with ablations of the SCN, functional properties of putative oscillators outside the SCN could be studied in isolation. Eight of 27 operated wild-type mice failed to display circadian rhythm in locomotor activity during *ad libitum* food access prior to the commencement of RF schedules (Fig. 1F). Seven of these mice were judged to have complete SCN ablations upon histological examination. The sequence of feeding conditions was as follows: *ad libitum* feeding for 3 days, no food for 30 h, 24-h RF for 8 days, no food for 25 h, *ad libitum* feeding for 87 h, no food for 30 h, 23-h RF for 15 days, 22-h RF for 12 days, 21-h RF for 10 days, 20-h RF for 11 days (Fig. 1B). The actograms indicated robust FAA in all mice exposed to a 24-h RF schedule after *ad libitum* feeding. The FAA phase estimated with circular analysis for a group was −1.4 h. The FAA was noticeably associated with feeding time when the feeding time was delayed by 6 h on the last day of the 24-RF schedule. The absence of free-running activity resulting from SCN ablation enabled us to quantify the presence of FAA (Fig. 1G), and the FAA ratios exceeded 2.0 in all 7 mice (FAA ratio: 4.06±0.85, n = 7). Comparable to the 24-h schedule, under the 23-h RF schedule, the mean activity profile indicated high levels of FAA (ratios >2.0) in 5 out of 7 mice and lower levels of FAA in 2 mice (mean FAA ratio: 2.78±0.68, n = 7). The mean activity profile indicated FAA at a phase of −0.9 h. In contrast to the 24- and 23-h RF schedules, the actograms indicated little or no FAA in mice on the 22-, 21- and 20-h RF schedules. Although 2 out of 7 mice on the 22-h schedule showed persistence of the FAA pattern, their FAA ratio was not significantly different from that during *ad libitum* feeding (1.78±0.43 vs. 0.88±0.15, n = 7, ANOVA post-hoc Dunnett test, *P* = 0.294). No obvious FAA was observed in mice on the 21- and 20-h schedules. In contrast to the FAA ratio, no significant difference was detected in either the feeding time activity ratio or the post-feeding activity ratio in each T-cycle (Fig. S1). These results suggest that the lower limit of entrainment to periodic feeding in wild-type mice is approximately 23 h and that the FAA rhythms are regulated by a circadian oscillator distinct from the SCN.

### Limits of Entrainment to Periodic Feeding in *Cry1*- or *Cry2*-deficient Mice with SCN Ablations

**Figure 2:**
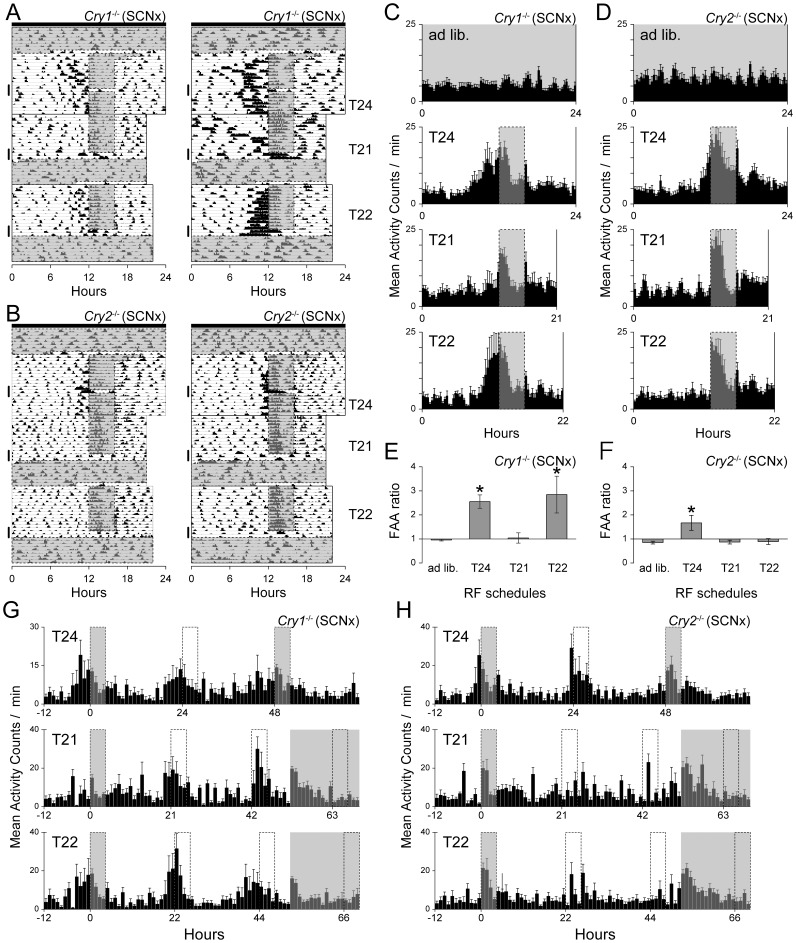
Limits of entrainment of FAA in *Cry1-* or *Cry2*-deficient mice. Representative actograms of locomotor activity plotted at a day length corresponding to each feeding schedule in *Cry1* ^−/−^ (**A**) and *Cry2* ^−/−^ (**B**) mice with SCN ablation. Time is plotted left-to-right in 10-min bins. Mice were maintained in constant darkness as indicated by closed bars at the top of the actograms. Feeding time is indicated by gray shaded boxes. (**C** and **D**) Mean profiles of locomotor activities are plotted along each RF schedule (**C**: *Cry1* ^−/−^, n = 8; **D**: *Cry2* ^−/−^, n = 7). Mean profile was calculated using individual mean 7-day profiles. Each data point represents counts per minute averaged across a 20-min bin. (**E** and **F)** FAA ratios of *Cry1* ^−/−^ (**E**) and *Cry2* ^−/−^ (**F**) mice on serial feeding schedules. Significant differences are indicated by asterisks (**E**: *Cry1* ^−/−^, n = 8; **F**: *Cry2* ^−/−^, n = 7, vs. *ad libitum*, ANOVA post-hoc Dunnett test). *P<0.05. (**G** and **H**) Mean serial plots of locomotor activity for 82 h represented in a 60-min bin. The last feeding time is set to zero. *Cry*-deficient mice were food-deprived for 44 h during the 24-h RF schedule (**G_T24** and **H_T24**), then returned to a 24-h RF schedule and food-deprived for 48 h after 21-h (**G_T21** and **H_T21**) and 22-h (**G_T22** and **H_T22**) RF schedules, then returned to *ad libitum* feeding. Serial-plotted portions are indicated by a thick vertical line in (**A**) and (**B**).

Mice homozygous for a null *Cry1* allele show a free-running period approximately 1 h shorter than in wild-type mice, whereas *Cry2*-null mice exhibit free-running periods of approximately 1 h longer than those seen in wild-type mice [23]. If the FEOs that regulate FAA rhythms depend on canonical clock genes, such circadian period mutations may affect the limits of the entrainment range to periodic feedings. To address this hypothesis, a paired group of *Cry1* ^−/−^ and *Cry2* ^−/−^ mice with SCN ablations were subjected to the following sequence of RF conditions: *ad libitum* feeding for 7 days, no food for 30 h, 24-h RF for 11 days, no food for 44 h, 24-h RF for 7 days, 21-h RF for 11 days, no food for 48 h, *ad libitum* feeding for 7 days, 22-h RF for 13 days, no food for 48 h, then *ad libitum* feeding (Fig. 2A and 2B). Among the 50 mice that underwent surgery (30 *Cry1* ^−/−^ and 20 *Cry2* ^−/−^), 8 *Cry1* ^−/−^ and 7 *Cry2* ^−/−^ mice showed a cessation of free-running activity rhythms under baseline conditions of *ad libitum* food, and were judged to have complete SCN ablations upon histological examination.

Under the 24-h RF schedule, 7 of 8 *Cry1* ^−/−^ mice and 5 of 7 *Cry2* ^−/−^ mice exhibited robust FAA (Fig. 2C_T24 2D_T24). When food was removed for 44 h during a 24-h schedule, the FAA circadian rhythms persisted in both genotypes (Fig. 2G_T24, Cry1^−/−^, period: 22.1 h, R^2^: 0.49, *p*<0.005, Fig. 2H_T24, Cry2^−/−^, period: 24.6 h, R^2^: 0.49, *p*<0.005).

When the periods of RF were shifted from the 24-h RF schedule to the 21-h RF schedule, all FAA disappeared (Fig. 2C_T21 and 2D_T21). A notable feature of the actogram is that, when the feeding schedule was shortened by 3 h, one *Cry1* ^−/−^ mouse showed persistence of an activity rhythm with circadian periodicity (23.4 h by chi-square periodogram, 23.3 h by least-squares fit to activity bouts; Fig. 2A right panel). No such circadian rhythms were observed in the *Cry2* ^−/−^ group. A second notable finding was that, when food was removed for 48 h at the end of the 21-h RF schedule, *Cry1* ^−/−^ mice exhibited a significant rhythm for a couple of cycles with activity bouts limited to around the time of the previous feeding (Fig. 2G_T21, 21.0 h, R^2^: 0.23, *p*<0.005). This result indicates that under the 21-h RF cycles, the entrained activity may fall in the 4-h feeding window, and may therefore not contribute to the quantification of FAA. No such circadian rhythms were observed during fasting in *Cry2* ^−/−^ mice (Fig. 2H_T21). These results suggest that the FEO in *Cry1* ^−/−^ mice could be entrained to a shorter period of T-cycle than could *Cry2* ^−/−^ mice.

This difference in the limits of entrainment to periodic feeding was apparent when each genotype was exposed to a 22-h RF schedule. Under the 22-h RF schedule, 6 of 8 *Cry1* ^−/−^ mice exhibited robust FAA (Fig. 2C_T22), whereas no *Cry2* ^−/−^ mice did so (Fig. 2D_T22). During a 48-h fast after a 22-h RF schedule, *Cry1* ^−/−^ mice showed persistent rhythms with an estimated period of 20.7 h (Fig. 2G_T22, R^2^: 0.53, *p*<0.005). Quantitative analysis of the FAA ratio revealed that *Cry1* ^−/−^ mice showed significant FAA in the 24-h and 22-h RF schedules (Fig. 2E), whereas *Cry2* ^−/−^ mice showed significant FAA only in the 24-h RF schedule (Fig. 2F). No significant difference was detected in either the feeding time or post-feeding activity ratios in each T-cycle (Fig. S2). These results indicate that the limits of entrainment to periodic feeding are dependent on genotype in *Cry*-deficient mice, further suggesting a dependence on the known molecular clock machinery.

### Non-oscillatory Properties of Pre-feeding Activity in *Bmal1*-deficient Mice

To establish the working hypothesis of the autonomous FEO, several studies, especially on rats, have revealed that once the FAA was established in a T = 24 h RF condition, it reappeared at the same time each day for at least 4 days following total food deprivation [26], [27]. This is reflective of the free-running rhythm of the FEO without periodic time cues. Another indication of the free-running rhythm of the FAA is that once FAA is established by RF, the return to *ad libitum* feeding conditions leads to the disappearance of the FAA. When rats are subsequently food-deprived, the behavioral activity reappears at the same circadian phase as previously temporally-restricted feeding [27], [28]. This suggests the persistence of the FEO with masking of FAA under *ad libitum* feeding conditions.

**Figure 3:**
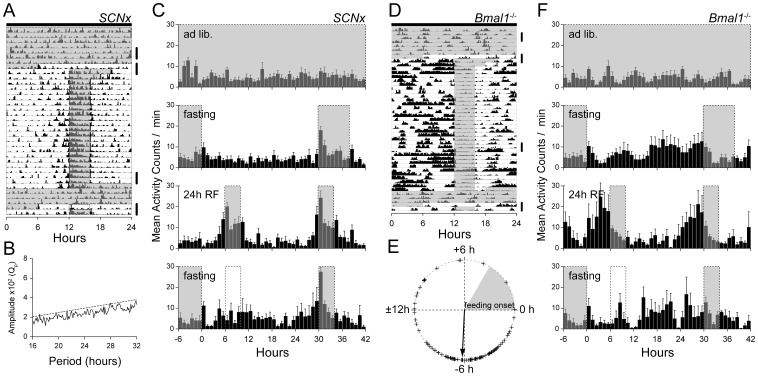
Persistence of FAA rhythms during food deprivation in the case of entrainment. (**A**) Representative actogram of locomotor activity in SCN-ablated C57BL/6 wild-type mice under 24 h RF schedule. Time is plotted left-to-right in 10-min bins. Mice were maintained in constant darkness as indicated by closed bars at the top of the actograms. Feeding time is indicated by gray shaded boxes. (**B**) Periodogram during the baseline before RF from the mouse represented in (**A**). (**C**) Group profiles of locomotor activity are plotted for 48 h in a 60-min bin (n = 6). Serial-plotted portions are indicated by a thick vertical line in (**A**). Previous feeding time is indicated by transparent dotted boxes. (**D**) Representative actogram of locomotor activity in *Bmal1* ^−/−^ mice under 24 h RF schedule. (**E**) Circular analysis of inter-day deviation of the pre-feeding activity phase in individual *Bmal1* ^−/−^ mice. The start of feeding time is set to zero. The mean pre-feeding activity phase to 24-h RF schedule is indicated by a thick arrow (n = 5). (**F**) Mean profiles of locomotor activity are plotted for 48 h in a 60-min bin (n = 6). Serial-plotted portions are indicated by a thick vertical line in (**D**).

Six wild-type mice with SCN ablations were maintained in constant darkness for at least 7 days and exposed to RF at periods of 24 h for 21 days (Fig. 3A). None of the mice displayed a circadian rhythm during ad libitum feeding (Fig. 3B and 3C_top*)* and the 30-h food deprivation period (Fig. 3C_2nd) before the RF schedule began. Mean activity levels did not differ between the *ad libitum* feeding period and the 30-h food deprivation period (Fig. 3C *_*top and 3C_2nd). The actograms and the mean activity profile indicated FAA in all mice exposed to a 24-h RF schedule with a steep increase of locomotor activity within 2 h before feeding time (Fig. 3C_3rd, pre-feeding activity phase: −1.55±0.42 h, n = 6). Pre-feeding activity deviation for the last 14 days of RF was 0.87±0.22 h (n = 6). After RF schedules, the mice were returned to *ad libitum* food access for 90 h following 30 h of food deprivation. During the second food deprivation period, mice exhibited increasing activity just before the previous feeding time (Fig. 3C_bottom). This activity profile was well fitted to a sine curve with the 24-h period peaking at the previous feeding time (R^2^: 0.20, *p*<0.05) suggesting the persistence of a once-established FAA rhythm.

To further explore oscillatory properties in molecular clock–deficient mice, we examined *Bmal1* ^−/−^ mice. *Bmal1* ^−/−^ mice failed to display circadian activity during *ad libitum* food access in DD (Fig. 3D and 3F_top). During 30-h food deprivation before the 24-h RF schedule, *Bmal1* ^−/−^ mice displayed growing, enhanced and ultradian patterns of activity (Fig. 3F_2nd). Five of 10 mice were removed from the progressive 24-h RF schedule when they were exhausted by continuous activity and took almost no food during the 4-h feeding time. The remaining 5 *Bmal1* ^−/−^ mice displayed FAA to a 24-h RF schedule with increasing locomotor activity (Fig. 3F_3rd, pre-feeding activity phase: −6.2±1.0 h, n = 5). Pre-feeding activity deviation for the last 20 days of RF was 3.3±1.7 h (n = 5), indicating large inter-day instability (Fig. 3E). After 32 days of 24-h RF, the mice were returned to *ad libitum* food access for 84 h following a 30 h food deprivation (Fig. 3F_bottom). During the second food deprivation, the activity profile of *Bmal1* ^−/−^ mice was similar to that before RF. Therefore, we could not conclude that the increasing activity before the previous feeding time corresponded to the persistence of an FAA rhythm, consistent with previous reports [16].

### Broader Range of Entrainment to Periodic Feeding in *Bmal1*-deficient Mice

**Figure 4:**
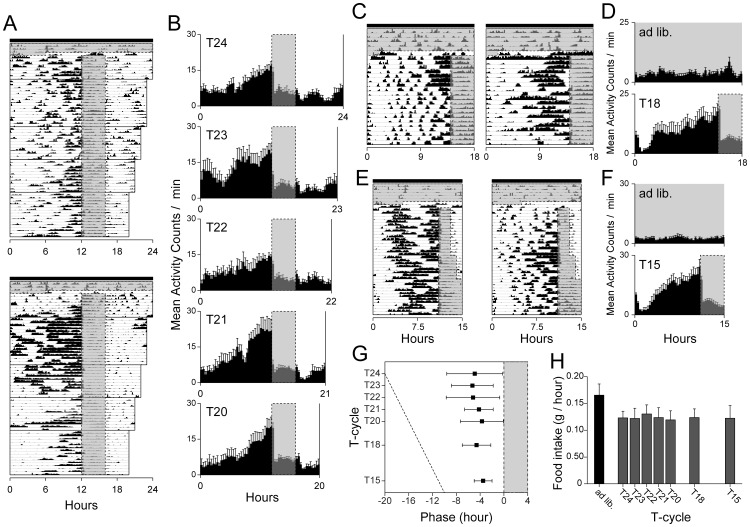
Broader range of pre-feeding activity rhythms in *Bmal1*-deficient mice. (**A**) Representative actograms of locomotor activity plotted at a day length corresponding to each feeding schedule in *Bmal1* ^−/−^ mice. Time is plotted left-to-right in 10-min bins. Feeding time is indicated by gray shaded boxes. (**B**) Mean profiles of locomotor activity are plotted along each RF schedule (n = 5). Mean profile was calculated using individual mean 7-day profiles. Representative actograms of locomotor activity plotted at a day length corresponding to 18-h (**C**) and 15-h (**E**) RF schedules in *Bmal1* ^−/−^ mice. Mean profiles of locomotor activities are plotted along 18-h (**D**, n = 7) and 15-h (**F**, n = 7) RF schedules in a 20-min bin. (**G**) Pre-feeding activity phases (circular analysis for individuals) to T-cycle schedules. Error bar indicates mean inter-day deviation of pre-feeding activity phases. (**H**) Food intake per hour during *ad libitum* and RF conditions.

To further explore oscillatory properties in *Bmal1* ^−/−^ mice, we exposed 10 *Bmal1* ^−/−^ mice for the following sequence of RF conditions: *ad libitum* feeding for 4 days, no food for 30 h, 24-h RF for 8 days, 23-h RF for 15 days, 22-h RF for 10 days, 21-h RF for 10 days, 20-h RF for 12 days. All mice had been exposed to the 24-h RF schedule for at least 2 weeks prior to the sequence, although 5 of the 10 *Bmal1* ^−/−^ mice were removed from the sequential T-cycles because of insufficient feeding. In contrast to wild-type and *Cry* KO mice, who showed a limited range of entrainment, *Bmal1* ^−/−^ mice exhibited an increase of activity in pre-feeding time under 24-, 23-, 22-, 21-, and 20-h feeding schedules (Fig. 4A and 4B). In the 18-h or 15-h RF conditions in another group of mice (n = 7), *Bmal1* ^−/−^ mice exhibited activity rhythms peaking at pre-feeding time (Fig. 4C–F). Thus, we did not observe a limited range of activity rhythm at T-cycle with any tested period (i.e., 15-h, 18-h, 20–24-h RF). Over the broad range of T-cycles, *Bmal1* ^−/−^ mice showed large inter-day instability of pre-feeding activity (Fig. 4G), although there was no significant difference among the pre-feeding activity phases (*p* = 0.16, Fig. 4G). During RF schedules, daily food intake decreased to 75% of *ad libitum* feeding levels and showed linear correlation to the period of T-cycles (Fig. 4H). As normalized by each period, there were no significant differences in food intake among T-cycle conditions (Fig. 4H). These results suggest that the pre-feeding activity rhythm in *Bmal1* ^−/−^ mice is controlled via time measurement that is outside of the circadian range.

## Discussion

### Circadian Oscillation of the FAA Rhythm – Dependence on the Known Molecular Clock

Our study confirms that restricted daily feeding schedules entrain a circadian oscillator located outside the SCN to regulate FAA rhythms, and that this basic mechanism depends on a molecular framework similar to the canonical clock model of transcription-translation feedback loops.

We demonstrated that *Cry1* ^−/−^ mice with SCN lesions can express FAA for a 22-h period, while *Cry2* ^−/−^ and wild-type individuals with similar lesions cannot. The proposed explanation is that this differential response is due to the respective periods of endogenous clockwork (*i.e.*, much shorter in *Cry1* ^−/−^ mice compared to *Cry2* ^−/−^ and wild-type mice). To go further with this strategy, it seems appropriate to expose these mice to long periods of food access (e.g., 25 or 26 h). The prediction is that only *Cry2* ^−/−^ mice, and eventually also wild-type animals, but not *Cry1* ^−/−^ mice, will express FAA with long T-cycles of food access. Most circadian periods in behavior, except in FAA, are determined by the intrinsic period of the master clock in the SCN [29], [30]. Certain clock gene mutations and deficits cause inherent short-period, long-period and arrhythmic phenotypes in behavior that occur in parallel within the SCN cells [31], [32], [33], as well as in other central and peripheral tissues [34], [35]. Although the FEO may not be identical to the SCN clock, the observation that *Cry1* ^−/−^ mice show a shorter period of FAA rhythm than *Cry2* ^−/−^ mice indicates that similar molecular components underlie the FEO and SCN. Studies of the T-cycle entrainable range appear to be an efficient approach not only for demonstrating an oscillatory property but also for exploring the intrinsic period of the circadian oscillation. Among several lines of clock gene–deficient mice, most exhibit persistence of FAA rhythm [13], [16], [17], [36], [37], although the *Per2^brdm^* mouse showed no FAA in a once-a-day feeding condition (24 h) [38]. In contrast, another line of *Per2*-deficient mice exhibited normal FAA [17]. It is possible that the apparently discrepant findings result from a difference in intrinsic period of FEOs in the two lines [39], [40], [41], [42]. Apparent differences in the circadian properties of clock gene–deficient mice will enable further investigation of the FEO.

### Pre-feeding Activity Rhythms in Bmal1-deficient Mice

A defining property of a circadian oscillator is the persistence of rhythmic output after the termination of periodic input. Recently, Mieda and Sakurai reported decreased but persistent FAA rhythm for 2 cycles after an RF schedule in nervous system–specific *Bmal1*-reduced mice [43]. In our monitoring of general locomotion in *Bmal1*-null mice, we failed to find persistence of pre-feeding activity rhythm comparable to that found previously in a study of wheel running activity under constant darkness [16]. These results indicate an essential role of *Bmal1* in regulating FAA rhythms as oscillatory output. In theory, oscillatory systems under forced periodicity show a limited range of entrainment in which autonomous oscillations with the period close to the period of the input cycle (T-cycle) are more likely to be entrained [44]. Therefore, results from *Bmal1* ^−/−^ mice under T-cycle indicate that the underlying mechanism controlling pre-feeding activity rhythms is not a (or a group of) robust oscillator(s). Recent studies mentioned the possibility that *Bmal2* could compensate the lack of *Bmal1* [45], [46], resulting “stochastic” or “damped” oscillation could be entrained to broader range of cyclic inputs. Lesion studies suggest the existence of a food-entrainable pacemaker outside the SCN; however, it has been reported that the RF entrains the SCN oscillators under certain conditions [12], [47]. Therefore, it cannot be fully excluded that the pre-feeding activity expressed by food-restricted *Bmal1* ^−/−^ mice results in part from de novo coupling between SCN cells that would become synchronized to the feeding Zeitgeber. In other words, the apparent pre-feeding activity in *Bmal1* ^−/−^ mice would actually be the locomotor activity rhythm controlled by the defective SCN more or less stabilized by daily feeding signals.

A common pattern of activity in *Bmal1* ^−/−^ mice is a cessation of activity during the first few hours after feeding, followed by a slow and gradual increase in the activity level over the remaining hours until the next feeding (Fig. 4). These pre-feeding activity patterns are distinct from the FAA profiles of both wild-type and *Cry* KO mice instead resembling conditioned behavior such as lever-pressing under RF schedules out of circadian range [18]. The consistent pre-feeding activity phase and food-intake rate of *Bmal1* ^−/−^ mice in every T-cycle (Fig. 4G and 4H) support the idea that an alternative timing mechanism drives pre-feeding activities without circadian regulation of the FEO. Recently, Luby *et al*. reported a pre-feeding activity rhythm in response to restricted feeding over a period of 18 h in C57BL/6J mice [48]. This discrepancy may be related to the procedures of restricted feeding. Their study reporting anticipation at intervals below the lower limit of the circadian range restricted calorie intake to 60% of the *ad libitum* feeding conditions, whereas in our temporally food-restricted wild-type mice, which had a food intake of 75% of the *ad libitum* level, we failed to observe FAA to 21- and 20-h T-cycles. These observations suggest that the pre-feeding activity in *Bmal1* ^−/−^ mice, independently of the periods of feeding cycles, may be dependent on more subtle non-circadian behavioral responses triggered by food deprivation. The interaction between a “long-interval timing” and the FEO remains to be elucidated [49]. Molecular clock–deficient mice provide crucial evidence for the involvement of molecular clock genes in circadian regulation of behavioral FAA, and they may be important models for furthering our understanding of behavioral regulation by interaction between time measurements and circadian rhythms.

## Supporting Information

Figure S1 **Activity ratios of the SCN-ablated mice on serial feeding schedules.** Feeding-time and post-feeding activity ratios were calculated by the fold change of mean locomotor activity in each mouse during the 4-h feeding period and the 2-h post-feeding period, respectively, and were compared with those during the rest of the day. No significant difference was detected in either the feeding-time activity ratio (**A**: n = 7, ANOVA, *P* = 0.88) or the post-feeding activity ratio (**B**: *P* = 0.31) in each T-cycle.
(TIF)

Figure S2 **Activity ratios of the SCN-ablated** ***Cry1*** **^−/−^ (A,C) and** ***Cry2*** **^−/−^ (B,D) mice on serial feeding schedules.** Feeding-time and post-feeding activity ratios were calculated in SCN-ablated *Cry1* ^−/−^ and *Cry2* ^−/−^ mice in a similar way as described in the legend of Figure S1. No significant difference was detected in either the feeding-time (**A**: *Cry1* ^−/−^, n = 8, ANOVA, *P* = 0.68; **B**: *Cry2* ^−/−^, n = 7, *P* = 0.85) or post-feeding (**C**: *Cry1* ^−/−^, n = 8, *P* = 0.70; **D**: *Cry2* ^−/−^, n = 7, *P* = 0.16) activity ratios in each T-cycle.
(TIF)
