# Supplementary figures and images for: Circadian Regulation of Food-Anticipatory Activity in Molecular Clock–Deficient Mice
Source: PLoS One. 2012 Nov 7;7(11):e48892. doi: 10.1371/journal.pone.0048892 (PMC3492221; doi:10.1371/journal.pone.0048892)

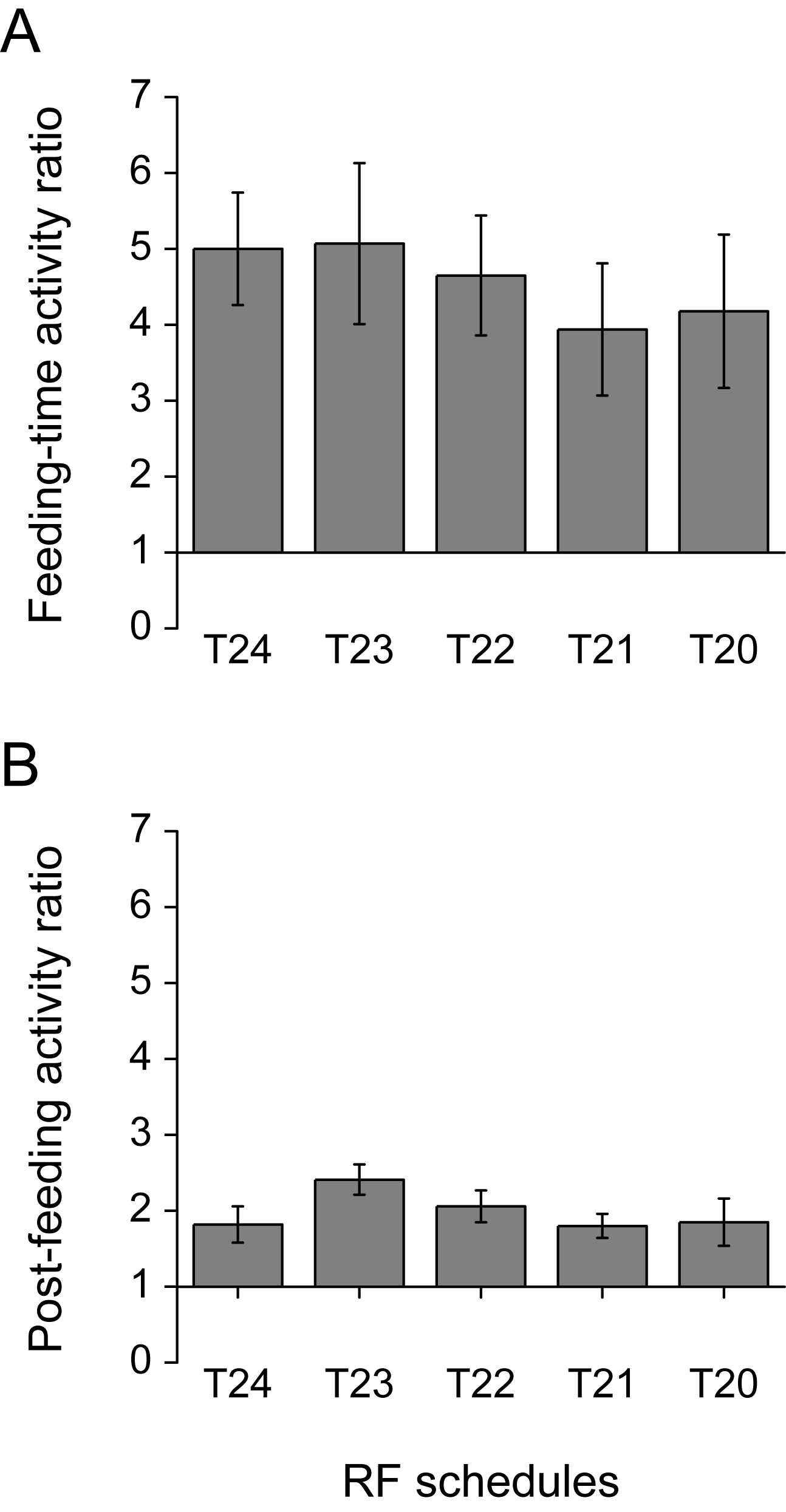

Supplement: Figure S1 — Activity ratios of the SCN-ablated mice on serial feeding schedules. Feeding-time and post-feeding activity ratios were calculated by the fold change of mean locomotor activity in each mouse during the 4-h feeding period and the 2-h post-feeding period, respectively, and were compared with those during the rest of the day. No significant difference was detected in either the feeding-time activity ratio (A: n = 7, ANOVA, P = 0.88) or the post-feeding activity ratio (B: P = 0.31) in each T-cycle. (TIF) [file pone.0048892.s001.tif]

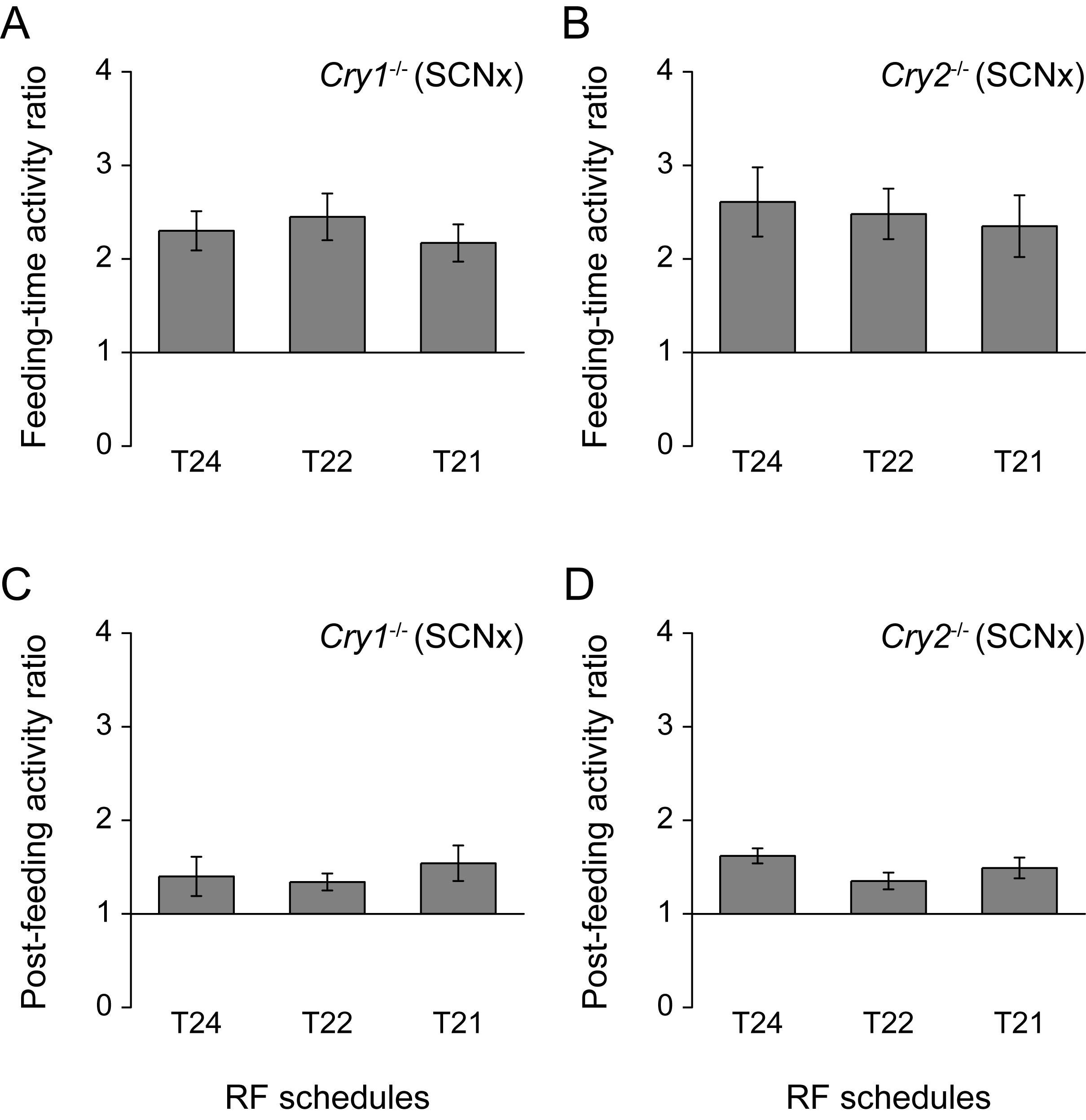

Supplement: Figure S2 — Activity ratios of the SCN-ablated Cry1 −/− (A,C) and Cry2 −/− (B,D) mice on serial feeding schedules. Feeding-time and post-feeding activity ratios were calculated in SCN-ablated Cry1 −/− and Cry2 −/− mice in a similar way as described in the legend of Figure S1. No significant difference was detected in either the feeding-time (A: Cry1 −/−, n = 8, ANOVA, P = 0.68; B: Cry2 −/−, n = 7, P = 0.85) or post-feeding (C: Cry1 −/−, n = 8, P = 0.70; D: Cry2 −/−, n = 7, P = 0.16) activity ratios in each T-cycle. (TIF) [file pone.0048892.s002.tif]
